# There Is an “Eye” in Team: Exploring the Interplay Between Emotion, Gaze Behavior, and Collective Efficacy in Team Sport Settings

**DOI:** 10.3389/fspor.2020.00018

**Published:** 2020-03-06

**Authors:** David A. Shearer, Shona Leeworthy, Sarah Jones, Emma Rickards, Mason Blake, Robert M. Heirene, Mike J. Gross, Adam M. Bruton

**Affiliations:** ^1^School of Psychology and Therapeutic Studies, University of South Wales, Treforest, United Kingdom; ^2^Welsh Institute of Performance Science, Swansea, United Kingdom; ^3^Sport and Exercise Science Research Centre, Department of Life Sciences, Whitelands College, University of Roehampton, London, United Kingdom; ^4^Brain and Mind Centre, School of Psychology, University of Sydney, Sydney, NSW, Australia

**Keywords:** team confidence, emotional contagion, group behavior, sport, vision

## Abstract

Little is understood about the attentional mechanisms that lead to perceptions of collective efficacy. This paper presents two studies that address this lack of understanding. Study one examined participant's (*N* = 59) attentional processes relating to positive, neutral, or negative emotional facial photographs, when instructed to select their “most confident” or “least confident” team. Eye gaze metrics of first fixation duration (FFD), fixation duration (FD), and fixation count (FC) were measured alongside individual perceptions of collective efficacy and emotional valence of the teams selected. Participants had shorter FFD, longer FD, and more FC on positive faces when instructed to select their most confident team (*p* < 0.05). Collective efficacy and emotional valence were significantly greater when participants selected their most confident team (*p* < 0.05). Study two explored the influence of video content familiarity of team-based observation interventions on attentional processes and collective efficacy in interdependent team-sport athletes (*N* = 34). When participants were exposed to familiar (own team/sport) and unfamiliar (unknown team/sport) team-based performance video, eye tracking data revealed similar gaze behaviors for the two conditions in terms of areas of interest. However, collective efficacy increased most for the familiar condition. Study one results indicate that the emotional expressions of team members influence both where and for how long we look at potential team members, and that conspecifics' emotional expression impacts on our perceptions of collective efficacy. For Study two, given the apparent greater increase in collective efficacy for the familiar condition, the similar attentional processes evident for familiar and unfamiliar team footage suggests that differences in meaning of the observed content dictates collective efficacy perceptions. Across both studies, the findings indicate the importance of positive emotional vicarious experiences when using team-based observation interventions to improve collective efficacy in teams.

Collective efficacy (Bandura, 1982) is a situational specific team confidence that increases team performance (Heuzé et al., 2006) and is important in socials domains such as education (Baker, 2001; Tasa et al., 2007), the military (Jex and Thomas, 2003), and business (Gibson, 2003) where successful domain specific outcomes rely on teamwork. In sport settings, the construct has been examined in volleyball (Fransen et al., 2012) football (Hampson and Jowett, 2014), wheelchair basketball (Shearer et al., 2009a), and adventure racing (Edmonds et al., 2009) where it is generally shown to have a positive impact on performance and group function. Sport is an ideal environment to study collective efficacy as most athletes compete in teams or groups (e.g., a training group), with fixed numbers, clear performance indicators, and work toward zero-sum goals (i.e., win/loss) (Widemeyer et al., 2002).

Mastery experiences are the most powerful antecedents of collective efficacy (Chase et al., 1997; Feltz and Lirgg, 1998; Myers et al., 2004), but the social dynamics of collective efficacy means vicarious experiences (i.e., observing team and non-team members) are also important (Goddard et al., 2000). Vicarious experiences can be manifested via imagery and observation interventions, where participants image, or observe team-related content (Shearer et al., 2009b; Bruton et al., 2016a). For example, Bruton et al. (2014, 2019) demonstrated how observation interventions enhanced collective efficacy in laboratory and applied experimental settings. In Bruton et al. (2014) first study, they demonstrated that positive observation interventions led to increased collective efficacy compared with neutral or negative interventions. In a second study, it was shown that collective efficacy increased regardless of whether participants observed their own team or another team, with the greatest increase occurring after observation of their own team performing. These results were extended by the same authors (Bruton et al., 2019) who found the use of observational learning interventions predicted collective efficacy (study one), and could be used to enhance collective efficacy in university level sports students (study two), and elite academy rugby players (study 3). However, despite these findings it is not yet clear what social information sources team members visually attend to when making these judgements of their team. Social cognitive mechanisms of the mirror neuron system and cortical midline structure (see Bruton et al., 2016a) suggest that this process involves emotional empathy (i.e., understanding how team members feel by observing their emotional display) and action observation and understanding (i.e., observing what their team mates do), but this has not been explored directly in the context of collective efficacy.

During social interactions our emotional states are revealed to those around us via expressions and non-verbal behaviors. When we observe others, we naturally mimic their facial expressions which helps us to understand their emotional experience at that moment (Buck, 1980). This tendency to mimic the emotional, motoric, sensory, and activation states of others is referred to as emotional contagion (Hatfield et al., 1994) and is suggested to function as the precursor to empathy (Prochazkova and Kret, 2017). From an empathy perspective, “automatic mimicry” of emotions is important for overall team function (McPherson et al., 2001) and evidence suggests team members are more likely to have positive emotional states, if they perceive teammates are in a good mood (Totterdell, 2000). These concepts are useful background for understanding the potential person-to-person transfer of social cognition information (including collective efficacy), as outlined in a recent model of emotional contagion.

The *Neurocognitive Model of Emotional Contagion* (NMEC, Prochazkova and Kret, 2017) provides a perception-action matching explanation of how emotional social signals (like collective efficacy) of a “sender” are transmitted via facial displays to a “receiver.” The model proposes that when a sender experiences an emotion (e.g., happiness) this results in subconscious autonomic (e.g., blushing) and motoric (e.g., smiling) responses which are visible to the receiver. Through a process of autonomic and motoric mimicry, perceptual inputs visible to the receiver allow for emotional understanding and a coupling of neural processes between both sender and receiver. Specifically, neural systems normally activated in the receiver when they feel happy simulate the affective state of the sender (Wood et al., 2016). The mirror neuron system (known to play a role in emotional contagion and facial mimicry), the limbic system (associated with empathy) and the anterior insula are all proposed to be active during this simulation process (Carr et al., 2003). While currently untested, the NMEC model provides an evidence-based explanation of how members of the same team transmit and receive information regarding their emotional states, which in turn influence individual perceptions of collective efficacy. Indeed, in the case of the limbic system Prochazkova et al. suggest that this brain area is essential for processing vicarious experiences (Kleckner et al., 2017), a known antecedent of collective efficacy (Bandura, 1986).

Social Cognitive Theory (Bandura, 1986) suggests individuals learn social behaviors through observation of others. Given that peer-modeling (i.e., observing) improves self-efficacy (Clark and Ste-Marie, 2002), team athletes may gain team mastery experiences, and more traditional vicarious experiences when they compare their own teams' performance to those of another team (e.g., a rival team). There is a growing body of research examining the benefits of action observation on motor and sport performance (McNeill et al., 2019, 2020). However, from a mechanistic perspective, the eye gaze and neuroscience evidence suggests that our capacity to understand and predict others' movements is directly tied to our own motoric knowledge of that action and an embodiment of the “observed person's” movement (Gredebäck and Falck-Ytter, 2015). The mirror neuron system (Rizzolatti et al., 2001) is activated during action understanding and reflects a visuo-motor matching process between what is “seen” and actions already “known” by the observer (Flanagan and Johansson, 2003). In the context of collective efficacy this matching might reflect, for example, how an individual appraises improvements or reductions in group function on the basis of observed team plays.

Given the primarily visual basis of understanding emotions and motor behavior in others, and the notion that this forms the basis of collective efficacy development, understanding eye gaze behavior in team setting is important for the future advancement of knowledge. Eye tracking is often used to explore relationships between visual attention and cognitive processes that precede superior performance and skill execution (e.g., Moran, 2009; Panchuk et al., 2019). Fixations are the most common measure of gaze behavior, and are classified in terms of duration, location and latency (McCormick et al., 2013). These metrics can be used to analyse conscious cognitive processes associated with visuomotor tasks (McCormick et al., 2013). However, despite the central role of eye gaze in social processes (Itier and Batty, 2009; Nummenmaa and Calder, 2009), little research has examined gaze behavior in complex social interactions (e.g., team sports). Based on the proposition individuals develop collective efficacy perceptions through observation of emotions and actions of teammates and other teams (Bruton et al., 2014, 2016b, 2019), eye gaze registration can be used to enhance our understanding of how bottom-up processing of salient information sources is of primary importance for the more top-down processing involved in collective efficacy judgements.

In this paper we outline two consecutive studies which examined gaze behaviors related to emotional recognition in teammates, and team-mastery and vicarious experiences. In study one, participants chose team-mates from a selection of passport-style headshot photographs depicting a range of emotions. Using eye tracking technology, used previously in emotion recognition research (Bal et al., 2010), we examined specific eye gaze metrics, collective efficacy perceptions, and the overall emotional valence of the team chosen when participants were instructed to select their “most” or “least” confident team. We hypothesized that when asked to choose their most confident team, participants would (a) fixate for longer (first fixation duration and fixation duration) and more often (fixation count) on positive emotional images, (b) have greater expectation of collective efficacy, and (c) select teams with greater aggregated positive emotional valence compared to their equivalent least confident team selection. In study two, we explored gaze behavior underpinning collective efficacy development in team athletes by examining how fixation metrics differed dependent on whether participants observed video content containing team mastery experiences (footage of own team) vs. traditional vicarious experiences (footage of non-familiar team). For “own team” footage, it was hypothesized participants would fixate for longer (fixation duration) and more often (fixation count) on the home team (i.e., team mastery experiences) compared to the away team (i.e., vicarious experiences) when judging collective efficacy. For the unfamiliar video footage, it was hypothesized individuals would fixate similarly on the home team and away team (i.e., vicarious experiences) as both were unknown to the participant. Finally, due to the combination of mastery and vicarious experiences available in the familiar condition, it was hypothesized that collective efficacy would increase most in this condition (Bruton et al., 2016a, 2019).

## Study One

### Method

#### Participants

Participants (*N* = 59) were an opportunity sample of undergraduate students, postgraduate students, and staff members from a UK university. The sample included male (*n* = 13: mean age: 22.76, SD: 2.71) and female (*n* = 46; mean age = 23.29, SD = 7.94) participants with ordinary or corrected-ordinary vision. Participants played a diverse range of sports (*n* = 19), with nearly half (*n* =27) not specifying a sport.

#### Materials and Measures

##### The NIMSTIM facial expression database (Tottenham et al., 2009)

The NIMSTIM facial stimulus set comprises 646 photographs of facial expressions designed for the study of emotion recognition. Nine emotions are portrayed with seventy different adults, and for our study, 150 unique photos were selected, representing a balance of positive (exuberant, happy, surprised, calm), negative (sad, fearful, disgusted, and angry) and neutral emotions. Before the study began these photos were scored on a scale of −10 (very negative emotional state) to +10 (very positive emotional state) by four members of the research team and the mean score across the different raters was used to dictate the “valence” and subsequent classification of the photo. During the study, a total emotional valence score was calculated based on the photographs participants selected for their team.

#### Obstacle Course Video

Participants were shown a third person perspective video of 3 unknown age-matched, and gender-mixed participants (i.e., 18–25 year olds) completing a gym-based obstacle course relay, which required teammates portrayed in the video to navigate the course holding a golf ball on a spoon. After each of their respective laps, team members transferred the golf ball to their blindfolded teammates using only the spoons. Participants in this study were led to believe they would be taking part in the obstacle course following the team selection task and that their selections would be used to pair them with the best possible teammates (see procedure).

#### Tobii Eye Tracking System

A Tobii pro TX120 (Tobii Technology) was used to measure eye movements during presentation of stimuli. The device consisted of a static screen-based eye tracker incorporated into a 17-inch monitor. The system uses a camera with infrared diodes to map reflection patterns on the corneas of the subjects' eyes, allowing measurement of fixations and saccades at a sample rate of 120 Hz. The Tobii eye tracking system was selected due to its high-level accuracy while allowing free head movement (Hirshkowitz and Wilcox, 2013). Participants were sat with their eyes 60 cm from the screen. Gaze behaviors recorded during intervention sessions were manually coded using “The Observer XT 11” computer software (Version: 11.5.718) in relation to the area they were located. Using minimum duration criterion consistent with previous eye tracking literature, any gaze point fixed on an area for more than 99.9 ms (twelve or more frames) within 2° of visual angle was classified as a fixation (McCormick et al., 2013). Any gaze point with a duration of 99.9 ms or less was classified as a “non-fixation” and discarded from the analysis.

First fixation duration (FFD), fixation duration (FD), and fixation count (FC) were measured in relation to participants eye gaze directed at the “areas of interest” (AOI) of positive, negative and neutral emotional expressions. All eye gaze measures were chosen to indicate which AOI drew the greatest attention in the context of the instructions given (see procedure below), and as an indirect marker of cognitive processing (Eckstein et al., 2017). FD provided a measure of the mean time each AOI was viewed. FC provided further detail as to whether the FD comprised of a single fixation on the AOI or multiple. With regards to FFD, as previous research has highlighted an early attentional bias toward threatening stimuli (Franklin et al., 2018) we used this measure to indicate whether the same was true in relation to negative facial expressions in the context of collective efficacy judgements. That is, do team member assess negative emotions in team mates as a threat to collective efficacy, and make choices on this basis.

#### Single Item Collective Efficacy Scale (Bruton et al., 2016b)

Bruton et al. (2016b) validated a single-item collective efficacy stem adaptable to different research and applied contexts. During validation, the item stem was compared to the Collective Efficacy Questionnaire for Sports [CEQS; (Short et al., 2005)] and was related to composite (β = 0.69) and the “ability” subscale (β = 0.51) scores for the CEQS, previous performance (β = 0.41), and three subscales (β *range* = 0.16–0.22) of the Group Environment Questionnaire (GEQ) (Carron et al., 1985). It also showed moderate concordance (pre-intervention; *r* = 0.53–0.74, post-intervention; *r* = 0.69–0.73) and good reliability (*r* = 0.77–088, 0.62–0.87) with the CEQS in two laboratory and field-based studies (Koo and Li, 2016). In this study, each time a participant selected a team from the facial photographs they were asked to respond to the following question: “*With you included, rate this team's confidence in their ability to perform to a high level, in order to achieve success on the obstacle course.”* This question was answered using a computer-based visual analog scale anchored with 0 (not confident at all) and 100 (completely confident). Participants recorded their response using the mouse pointer to click on the visual line at the point that indicated their belief at that moment. Collective efficacy was measured for each team that participants selected from the presented slides (i.e., 30 times) and a mean score calculated for the “most” and “least” confident conditions (based on 15 slides for each condition).

#### Single Item Self-Efficacy Scale

The single item collective efficacy scale was adapted to assess participant's level of self-efficacy before the team selection element of the experiment to control for individual differences in self-efficacy on collective efficacy (cf. Bruton et al., 2019). The item asked the individual to “*Rate your confidence in your ability to perform to a high level in order to achieve success on the obstacle course”* and record a response on a visual analog scale between 0 (not confident at all) and 100 (completely confident).

#### Procedure

Ethical approval was provided by the University of South Wales, Faculty of Life Science and Education Research Ethics Committee. Participants were provided with an information sheet that detailed the study, although the true nature of the study was withheld until after data collection was completed. Participants provided informed consent prior to taking part in the experiment.

Before the experiment began, participants were told that they would be required to select a team of three, consisting of themselves and two other strangers, that would compete against other university teams on a team-based obstacle course. They were informed that before they selected their final team, they would complete a team-selection experiment to determine suitable teammates. This manipulation was to ensure participants felt team selections were for a meaningful purpose and to maximize their engagement with the experimental task that followed.

Participants watched a video of the team obstacle course being completed successfully by strangers and completed the self-efficacy scale. Following individual calibration with the eye tracker, they read a set of instructions relating to the experimental procedure and were given a paper plan of the obstacle course in a visual birds-eye-view format. Participants were asked to consider the obstacle course task for each of the team selection choices made during the subsequent slides.

Prior to the experiment, each participant completed the manufacturer's calibration process for the eye tracking hardware. Following this, thirty slides were presented to each participant, each displaying five pre-rated faces with a range of emotional expressions. Each slide portrayed 1 extremely negative face (−7 to −10 rated), 1 moderately negative face (−3 to −6), 1 neutral face (−2 to 2), 1 moderately positive face (3–6) and 1 extremely positive face (7–10). Faces were presented in two rows, with three faces on the top row and two faces on the bottom row, and the position of the different emotional expressions were randomly ordered for each slide. Specific instructions alternated slide-by-slide, asking participants to either select the most confident team (15 slides) or the least confident team (15 Slides). Participants selected two people from each slide, verbally stating the unique code for each face. Each slide was presented for 10 s and between selections participants were asked to rate the team's collective efficacy for the obstacle course task. Post-experiment, all participants were debriefed regarding the true nature of the study and told they would not be physically completing the obstacle course task.

#### Data Analysis

Data analysis was completed using R Studio (version 1.1.383). Eye gaze data was examined using 3 separate multi-level models with FFD, FD, and FC as dependent variables and “participant” as a random effect. For each dependent variable a baseline model was created, against which 3 further models were compared. The models consisted of the main effects for “instruction” (i.e., least confident v most confident) and “AOI” (positive v negative v neutral), and then a final interaction model (instruction v AOI). Two *post-hoc* orthogonal contrasts were completed to examine the nature of significant interactions. For each multilevel model, contrast one examined the combined effects of all positive and negative images relative to neutral images when comparing the effects of participants being instructed to select either their least or most confident team. Contrast two examined the effects of positive images relative to negative images when comparing least and most confident groups. A repeated measures MANCOVA was employed to examine the differences in mean collective efficacy and emotional valence scores between the most confident and least confident condition while controlling for pre-experimental self-efficacy.

## Results

### First Fixation Duration

**Figure 1:**
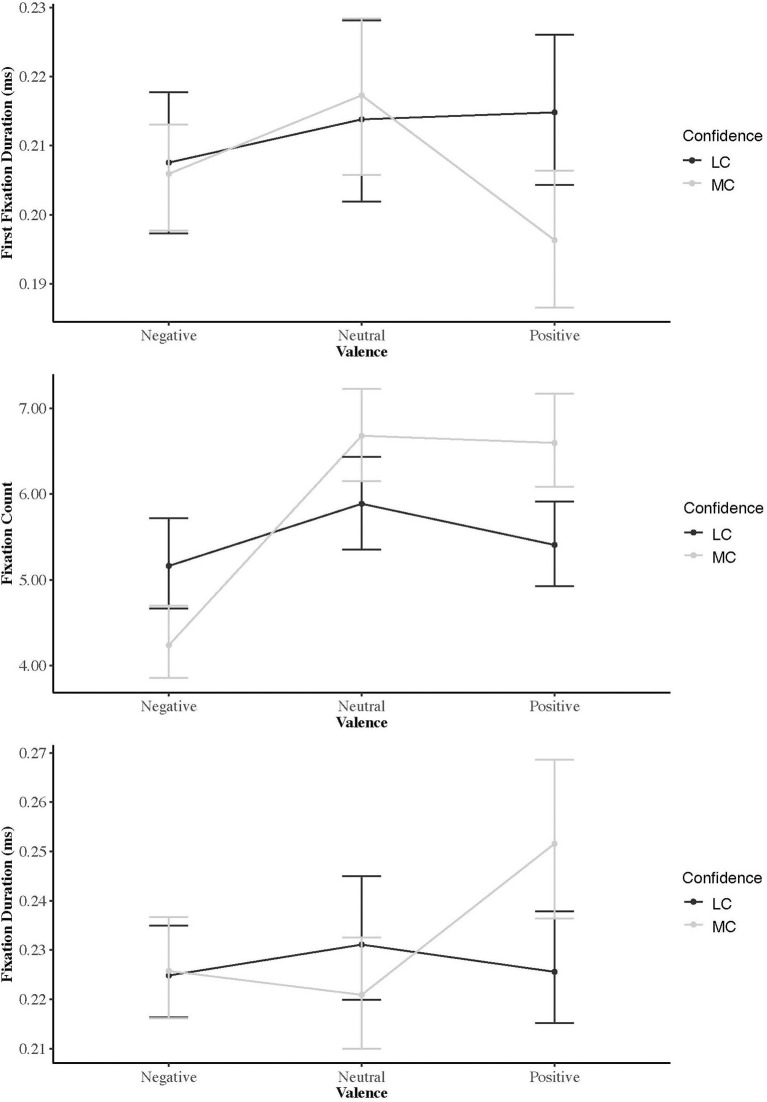
From Study 1: Eye gaze measures as a function of the emotional valence of the presented facial expressions.

FFD differed as a function of AOI [χ^2^_(2)_ = 12.87, *p* < 0.01], instructions [χ^2^_(1)_, = 5.08 *p* < *0*.01], and the interaction of both conditions [χ^2^_(2)_ = 15.23, *p* < *0*.001]. Orthogonal contrast indicated combined scores for positive and negative images differed from neutral images as a function of instructions given [*b* = 0.003, *t*_(232)_ = 3.82, *p* < 0.001, *r* = 0.24], but there was no significant difference between positive and negative imagery as a function of instruction [*b* = 0.001, *t*_(232)_ = 0.87, *p* = 0.38, *r* = 0.05]. Descriptive statistics and visual inspection of the data (Table 1, Figure 1) indicated that the significant interaction was a function of FFD for positive images in the most confident condition being shorter compared to all other conditions. Multiple comparisons with Bonferroni corrections confirmed most confident—positive was the only variable that differentiated between instructions (*p* < 0.001). Within the most confident condition, FFD for positive images was significantly less than both neutral (*p* < 0.001) and negative images (*p* < 0.05). All other comparisons were non-significant (*p* > 0.05).

**Table 1:** Descriptive statistics for study one.

**Confidence**	**Valence**	**FFD mean**		**FC mean**		**FD mean**	
		**Mean**	**SD**	**Mean**	**SD**	**Mean**	**SD**
MC	Negative	0.21	0.03	4.24	1.66	0.23	0.04
MC	Positive	0.20	0.04	6.60	2.12	0.25	0.06
MC	Neutral	0.22	0.05	6.68	2.15	0.22	0.04
LC	Negative	0.21	0.04	5.16	2.04	0.23	0.04
LC	Positive	0.21	0.04	5.41	2.00	0.22	0.05
LC	Neutral	0.21	0.05	5.89	2.17	0.23	0.05

*For “Confidence” column MC, Most confident; LC, Least confident. Valence column represents the emotional expressions displayed on the faces on each slide. FFD, First fixation duration; FC, Fixation count; FD, Fixation duration*.

### Fixation Count

FC differed as a function of AOI [χ^2^_(2)_ = 129.0, *p* < 0.001], instructions [χ^2^_(1)_, = 10.78, *p* < 0.001] and the interaction of both conditions [χ^2^_(2)_ = 86.01, *p* < 0.001]. Orthogonal Contrasts indicated that combined scores for positive and negative images differed from neutral images as a function of instructions given [*b* = −0.20, *t*_(232)_ = −6.46, *p* < 0.001, *r* = 0.39], and a significant difference between positive and negative images as a function of instruction [*b* = 0.43, *t*_(232)_ = 7.65, *p* < 0.001, *r* = 0.44]. Descriptive statistics and visual inspection of the data (Table 1, Figure 1) indicated that the interaction between combined positive and negative scores compared to neutral scores was accounted for by difference between negative and neutral images for the most confident condition compared to the least confident condition. Subsequent pairwise comparison indicated participants looked at negative images less than both neutral (*p* < 0.001) and positive images (*p* < 0.001) in the most confident condition. In the least confident condition, there was only a significant difference between the negative and neutral condition (*p* < 0.001). Multiple comparisons with Bonferroni corrections indicated no difference between positive and negative images in the least confident group (*p* > 0.05), but participants fixated more often on positive images in the confident group (*p* < 0.001). Within both instruction conditions, comparisons indicated significant differences between the frequency participants fixated on each different AOI (*p* < 0.05–0.001), apart from neutral and positive images for the most confident condition, and positive and negative images for the least confident condition (*p* > 0.05).

### Fixation Duration

FD differed as a function of AOI [χ^2^_(2)_ = 28.58, *p* < 0.001], instructions [χ^2^_(1)_ = 5.36, *p* < 0.05], and the interaction of both conditions [χ^2^_(2)_ = 52.01, *p* < 0.001]. Orthogonal Contrasts indicated combined scores for positive and negative images differed from neutral images as a function of instructions given [*b* = −0.005, *t*_(232)_ = −7.22, *p* < 0.001, *r* = 0.42], and a significant difference between positive and negative images as a function of instruction [*b* = 0.002, *t*_(232)_ = −2.26, *p* < 0.05, *r* = 0.15]. Descriptive statistics and visual inspection of the data (Table 1, Figure 1) indicated the interaction between combined positive and negative scores compared to neutral scores was accounted for by differences between positive and neutral images for the most confident condition. Pairwise comparisons confirmed participants looked at positive images for longer than neutral images when instructed to select their most confident team (*p* < 0.001), whereas there was no significant difference between any of the image conditions when instructed to select their least confident team (*p* > 0.05). For the significant contrast between positive and negative images there was no difference between conditions in the least confident group (*p* > 0.05), but participants did look at positive images longer compared to negative images when instructed to select their most confident team (*p* < 0.001). This was confirmed by pairwise comparisons which indicated that participants fixated on positive images for significantly longer when instructed to select their most confident team compared to any other image type in either instruction condition (*p* < 0.05–0.001). All other within and between comparisons were non-significant (*p* > 0.05).

### Emotional Valence and Efficacy

**Figure 2:**
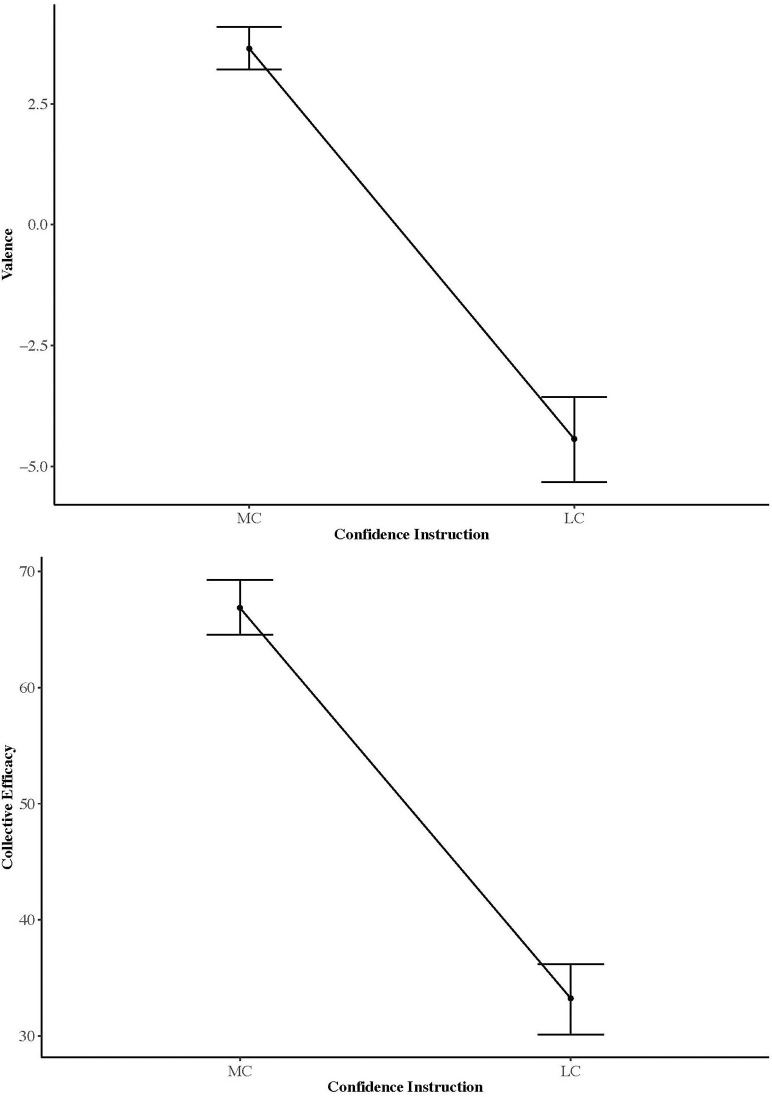
From Study 1: Emotional valence and collective efficacy scores as a function of most or least confident team selection.

Repeated measures MANCOVA indicated a significant overall main effect for instruction [*F*_(1,56)_ = 37.03, *p* < 0.001, η = 0.571], with a non-significant contribution from pre-experimental self-efficacy [*F*_(1,56)_ = 1.33, *p* = 0.27, η = 0.045]. Follow-up univariate tests indicated collective efficacy scores [*F*_(1,57)_ = 68.98, *p* < 0.001, η = 0.55] and emotional valence scores [*F*_(1,57)_ = 20.03, *p* < 0.001, η = 0.26] differed as a function of instruction, with mean scores indicating both collective efficacy and emotional valence scores were lower when participants were instructed to select their least confident team (Figure 2). Test of between subject effects indicated the effects of pre-experimental self-efficacy significantly and positively adjusted the relationship between collective efficacy and how participants were instructed to select their team [*F*_(1,57)_ = 16.4, *p* < 0*.0*01, η = 0.22], but did not significantly adjust the relationship with emotional valence scores and instructions given [*F*_(1,57)_ = 0.01, *p* = 0*.9*22, η = 0*.2*6].

## Study Two

### Method

#### Participants

An opportunity sample of 34 (Male = 19, Female = 15, *M*_*age*_ = 20.61, *SD*_*age*_ = 1.73) interdependent team-sport athletes from a UK university participated in this study. Participants competed at British Universities & Colleges Sport (BUCS) levels in men's football (*n* = 7), men's rugby (*n* = 4), men's basketball (*n* = 6), men's volleyball (*n* = 2), women's football (*n* = 10), and women's netball (*n* = 5).

#### Materials and Measures

##### Competitive team sports video

Performance video footage from two competitive fixtures per team was collected over 8 weeks. The videos were presented from a third-person perspective, as per the viewpoint of a spectator on the touchlines. The investigator positioned themselves at three points along the two respective touchlines lengthways (one quarter pitch/court, half pitch/court, and three quarters pitch/court) to record accurate footage of the different components of team performance in the sports. Video was edited into multiple clips displaying successful team performance (*M*_*clips*_ = 32 per team) using Windows Movie Maker (Version 2012, Build 16.4.3508.0205) at thirty frames per second. Eleven video clips, each lasting 12 s were selected for each team's video footage. The final videos included equal footage displaying successful performance (i.e., team skill execution, team scores), celebrations, and positive interactions between teammates. All squad members were included in at least four clips used for the team-based video. This meant that participants would observe themselves, as a member of the team, being involved in team performance in at least four clips.

#### Collective Efficacy Questionnaire for Sports (CEQS)

The CEQS (Short et al., 2005) was used to measure individual-level perceptions of collective efficacy. The CEQS is a 20-item collective efficacy measure that asks individuals to “*Rate your team's confidence in terms of upcoming competition, that your team has the ability to…”* on a 10-point scale ranging between 0 *(not at all confident)* and 9 *(completely confident)*. The CEQS has five factors that reflect ability, effort, persistence, preparation and unity. Scores can be produced for all factors, but studies tend to adopt a composite collective efficacy score based on the mean value for all questionnaire items (e.g., Bruton et al., 2014, 2016a). Confirmatory factor analysis by Short et al. (2005) indicated strong factorial validity for the CEQS [$\chi_{\left(160\right)}^{2}$ = 574.29, *p* < 0.001, NNFI = 0.90, CFI = 0.92, SRMR = 0.04, RMSEA = 0.09 (90% CI = 0.87–0.104)]. Strong internal reliability coefficients have been reported (α = 0.85–0.96) (Short et al., 2005; Bruton et al., 2014) and for this study, high Cronbach alpha scores for pre- (α = 0.97) and post-intervention (α = 0.97) were recorded.

#### Tobii Eye Tracking System

A Tobii X120 fixed eye-tracker running Tobii Studio was used to record gaze behavior during the intervention sessions (sampling rate of 120 Hz). Data processing was the same as study one, where only fixations on the areas of interest (AOI; home team, away team, ball) were selected for analysis as they represent team mastery and vicarious experiences, the strongest antecedents of collective efficacy beliefs (see e.g., Bruton et al., 2016b). Home AOI was defined as when the fixation was held on any body part of a member of the participant's own team (familiar condition) or the team representing the participant's university (unfamiliar condition). Away AOI was defined as when the fixation was held on any body part of a member of the participant's opposition team (familiar condition) or the team competing against the team from the participant's university (unfamiliar condition). Ball AOI was defined as when the fixation was held on the object that was central to the task being completed (i.e., football, rugby ball, basketball, or volleyball). To enhance reliability of the coding process, one research team member and a researcher not involved in the study independently coded gaze points for all video footage. Strong positive correlations between the two sets of coding data for number of fixations (*r* = 0.98–0.99, *p* = 0.00) and duration of fixations (*r* = 0.98–0.99, *p* = 0.00), legitimized the use of mean values for the two coders in the main analysis.

### Procedure

Ethical approval was granted by the University of Roehampton Research Ethics Committee. Participants provided written informed consent before filming of the video and participation in the experiment.

#### Experimental Design

A repeated-measures experimental design was used to examine the influence of familiarity with the team-based videos on collective efficacy and gaze behavior. Teams were paired in relation to gender ([1] Men's football—Men's rugby, [2] Men's basketball—Men's volleyball, [3] Women's netball—Women's football). Participants watched both familiar and unfamiliar team-based videos (counterbalanced) across two separate experimental sessions. Familiar videos consisted of footage of own team performance, while unfamiliar videos contained performance footage of the unfamiliar paired team.

#### Experimental Phase

Participants recorded collective efficacy using the CEQS before sitting at the eye-tracker. Eye tracker positioning and calibration was the same as for study one. Instructions for the experiment were presented on screen. The team-based video was presented as eleven separate clips using Tobii Studio. Immediately before each clip, participants were informed that they would be required to verbally rate their own team's collective efficacy after each clip. This was done to prime participants to observe with collective efficacy judgments in mind. After 7 days, participants returned to complete the second corresponding session mirroring the format of the first. Following each team-based video session, collective efficacy was recorded again using the CEQS. Upon completion of both video sessions, a brief semi-structured social validation interview was conducted with participants to gather their perceptions about the two conditions (Page and Thelwell, 2013). Questions related to perceived effects and information taken from the videos. Finally, participants were debriefed on the study aims and thanked for their involvement.

#### Data Analysis

Data analysis was completed using R Studio (version 1.1.383). Eye gaze data was examined using 2 separate multi-level models with FC and FD as dependent variables and “participant” as a random effect. For each dependent variable a baseline model was created, against which 3 further models were compared. The models consisted of the main effects for “AOI” (i.e., Home, Away, Ball) and “Familiarity” (Familiar and Unfamiliar), and then a final interaction model (AOI v Familiarity). *Post-hoc* orthogonal contrasts were completed to examine how the “Familiarity” condition led to differences in the AOI people viewed. Specifically, for each multilevel model, contrast one examined the combined effects of all “Home” and “Away” AOI compared to “Ball” AOI relative to the “Familiarity” condition (i.e., own team v different sport). Contrast two examined effects of “Home” vs. “Away” AOI relative to the “Familiarity” condition. For collective efficacy, a multilevel model was used to examine differences pre and post intervention in respect to “Familiarity.” A baseline model was created, against which 3 further models were compared. The models consisted of the main effects for “Familiarity” (i.e., Familiar and Unfamiliar) and “Timepoint” (Pre- and Post-intervention) and then a final interaction model. *Post-hoc* contrasts were used to examine the nature of any differences in collective efficacy in respect to the independent variables.

### Results

#### Number of Fixations

**Figure 3:**
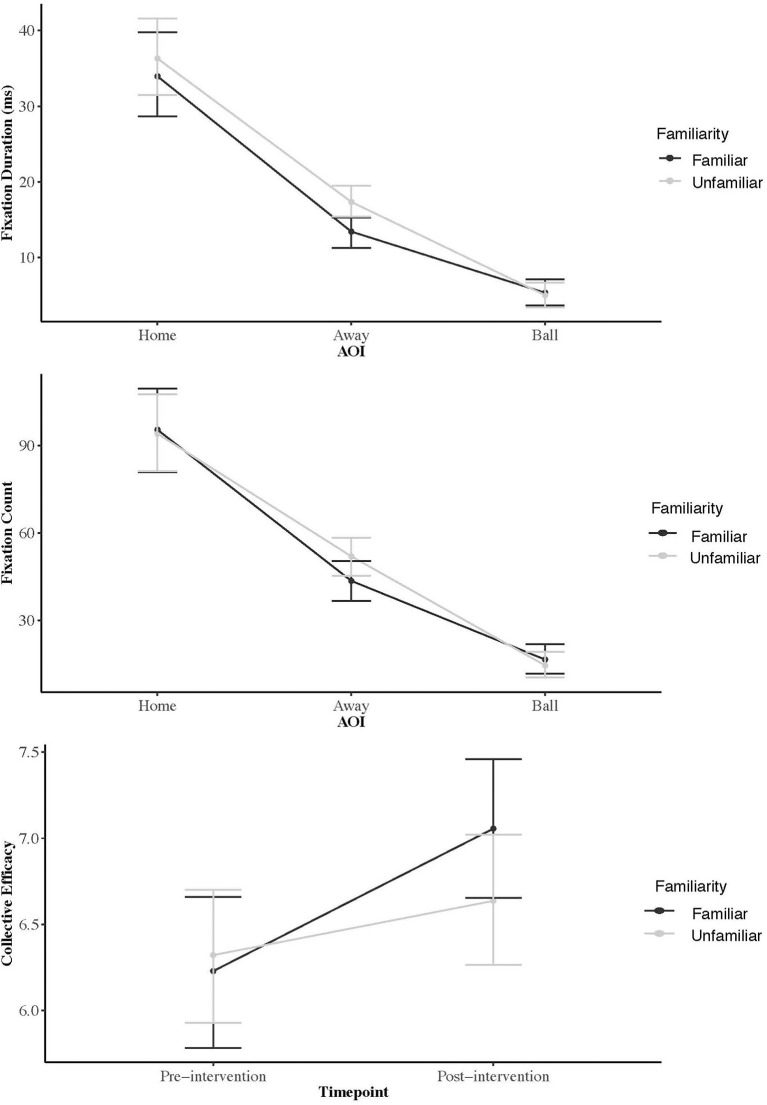
From Study 2: Eye gaze metrics as a function of the “Area of Interest” and collective efficacy scores pre- and post-intervention.

Compared to the baseline model FC differed as a function of AOI [χ^2^_(2)_ = 206.39, *p* < 0*.0*01], and orthogonal contrasts indicated participants looked less frequently at the ball compared to the home and away AOI combined (*t* = 16.84, *p* < 0.001, *r* = 0.82) and more frequently at the home vs. away team AOI (*t* = −12.30, *p* < 0.001, *r* = 0.83). No main effect was observed regarding Familiarity [χ^2^_(2)_ = 0.17, *p* = 0.67] and there was no significant overall interaction effect [χ^2^_(2)_ = 2.38, *p* = 0.30]. Examination of mean scores (Table 2, Figure 3) indicated that participants fixated for the same number of times on each AOI irrespective of the effects of the familiarity condition. Specifically, participants looked most often at the home team, followed by the away team, and then the ball.

**Table 2:** Descriptive statistics for study two.

**AOI**	**Familiarity**	**FC**		**FD**	
		**Mean**	**SD**	**Mean**	**SD**
Home	Familiar	95.44	44.56	33.96	17.18
Home	Unfamiliar	94.07	40.76	36.31	15.98
Away	Familiar	43.56	21.16	13.44	6.21
Away	Unfamiliar	51.97	19.31	17.36	6.17
Ball	Familiar	16.51	15.57	5.35	5.29
Ball	Unfamiliar	14.51	13.19	5.03	4.82

*AOI, Area of interest; Familiarity, experimental manipulation of either familiar or unfamiliar video footage; FC, Fixation count; FD, Fixation duration*.

#### Fixation Duration

Compared to the baseline model FD differed as a function of AOI [χ^2^_(2)_ = 192.42, *p* < 0.001, *r* = 0.82], and orthogonal contrast indicated participants looked less frequently at the ball compared to the home and away AOI combined (*t* = 14.62, *p* < 0.001, *r* = 0.78) and more frequently at the home AOI compared to away AOI (*t* = −12.45, *p* < 0.001, *r* = 0.73). However, no significant main effect was found for Familiarity [χ^2^_(2)_ = 1.84, *p* = 0.17] and there was no significant overall interaction effect [χ^2^_(2)_ = 1.86, *p* = 0.39]. Examination of mean scores (Table 2, Figure 3) indicated that participants fixated for the same amount of times on each AOI irrespective of the effects of the familiarity condition. Specifically, participants looked longer at the home team, followed by the away team, and then the ball.

#### Collective Efficacy

Compared to the baseline model collective efficacy did not differ significantly as a function of Familiarity [χ^2^_(2)_ = 0.98, *p* = 0*.3*2, *r* = 0.16], but was significantly different with respect to Timepoint [χ^2^_(2)_ = 36.29, *p* < 0*.0*01, *r* = 0.67] and there was a significant overall interaction effect between Familiarity and Timepoint [χ^2^_(2)_ = 10.40, *p* < 0*.0*01, *r* = 0.37]. Pairwise comparisons suggested there were no significant pre-interventions differences in collective efficacy between the familiar and unfamiliar conditions (*p* = 0.59). However, a significant difference was observed in post intervention collective efficacy score (*p* = 0.04) indicating that although collective efficacy increased after the videos for the unfamiliar condition, a greater increase was observed for the familiar condition (Figure 3). Even though differences in collective efficacy score pre-intervention were non-significant, some of the interaction effect is also explained by the cross over in collective efficacy, whereby scores were lower for the familiar compared to unfamiliar conditions at pre-intervention, but higher after the intervention.

#### Social Validation

Social validation data revealed all participants perceived familiar videos improved collective efficacy, while 61.8% of participants perceived unfamiliar videos benefitted collective efficacy. When asked why familiar videos had this effect, participants suggested it reminded them about positive aspects of their teams' performances (mastery experiences). For example, participant 16 stated “*I think it just validated like how I already feel about the team. Like we are very confident in our team and that we will succeed in any game we play”* and participant 22 commented “*it made me think more confidently about our team, I thought we were pretty good and watching it back it shows how well we can play.”* Participants who perceived the unfamiliar video as beneficial, indicated the footage allowed them to compare their team to the unfamiliar teams. For example, participant 19 said “*it made me more positive. You can see aspects that they do well and you think my team does that well, my team does this well, which highlights the good things.”* For participants who perceived the unfamiliar intervention had no effect, the main theme was the lack of transferrable aspects across the sports (model disparity). For example, participant 6 suggested that “*volleyball is probably a lot different from basketball so I couldn't really take anything apart from the effort they were putting in.”*

## Discussion

Taken together, both studies provide partial support that collective efficacy judgements are obtained, through the attentional process of observation, and the cognitive processing of visual information. Study one aimed to examine participants' preferences for teammates' emotional expressions in a novel team selection task. It was hypothesized that when instructed to select their most confident team, participants would (a) fixate more often and for longer on positive faces, (b) have greater expectation of collective efficacy, and (c) select a team with a greater aggregated positive emotional valence than when directed to select their least confident team.

For the most confident condition, results suggested FFD was significantly shorter for positive images, indicating participants looked at neutral or negative images for longer on immediate presentation of each slide. Overall however, participants fixated on positive images for longer (FD) than negative and neutral images, and more often (FC) than negative images. There was no difference in terms of how often (FC) people fixated on positive and neutral images, but they did look at negative images less. These differences in eye gaze metrics indicated that participants were taking longer to process information in positive pictures than either neutral or negative (Meghanathan et al., 2014), which in terms of collective efficacy might indicate they were trying to decide which positive teammate they would prefer in their team.

The disparity between FFD and FD for positive emotional faces reflects the time over which each slide was presented. The greater FFD for negative faces indicates an initial attentional bias toward threatening or aversive stimuli (Fashler and Katz, 2014; Duque and Vázquez, 2015), as it has previously been shown that angry (negative) faces are easier to detect than neutral or happy faces (Hansen and Hansen, 1988). While most research on attentional bias focusses on differences between anxious and non-anxious participants, even those with low anxiety have been shown to display an emotional bias when there is cumulative exposure of stimuli as was the case here (Bar-Haim et al., 2007). Indeed, an attentional bias toward threat related stimuli would likely form an important precursor to group-based perceptions in general. Therefore, we suggest that when instructed to select their most confident team, positive faces were immediately distinguishable, while neutral and negative faces required greater informational processing (i.e., “who do I not want in my team?”). Research indicates that manipulations of first fixations, do not ultimately affect the choices people make, and that total fixation duration (which does affect choice) is largely driven by the task instruction (van der Laan et al., 2015). In this instance therefore, as participants were (i) instructed to select their most confident team and, (ii) Social Identity Theory (Tajfel, 1974) suggests that people are more likely to surround themselves with positive people who maintain their own positive self-concept, it is not surprising that FD for positive faces was greater.

Although it was hypothesized FFD, FD and FC for negative images would be greater in the least confident condition, differences were only found for FC. Specifically, neutral images were fixated on more often than either positive or negative images. There is no clear reason for this finding, however, although not significant, a similar profile was observed for FD (Figure 1). Todorov et al. (2008) suggest that when evaluating emotional valence of neutral faces we look for subtle expressions that suggest whether there are negative or positive emotions underlying the expression. We therefore speculatively suggest neutral images required greater information processing in the least confident condition because the faces portrayed in the images were emotionless and ambiguous. This ambiguity would require more attention and therefore greater FC. Furthermore, Bandura (1986) suggests emotional arousal is a determinant of efficacy beliefs; with no emotional information, participants would take longer and fixate more often to ascertain the suitability of the neutral face. Emotional valence scores for the least confident condition indicate that even though neutral faces were visited more often, participants eventually selected negative emotional faces.

Overall, the eye gaze metrics in this study paint a consistent pattern regarding participants' preference for positive facial emotions when selecting confident teams. Previous research highlights the importance of the human face and emotions in gathering first impressions about people around us (Bar et al., 2006; Willis and Todorov, 2006). Barsade and Gibson (1998) emphasize the bottom-up development of group emotions, where non-verbal cues (e.g., facial expressions) are an important determinant of “emotional contagion.” The NMEC model (Prochazkova and Kret, 2017) provides a mechanism for how we understand and reflect others' emotions, simply by observing physiologic and motoric aspects of people's faces. Due to the nature of the still images used in our study, the underlying physiology of the faces portrayed could not be judged by participants. However, motoric aspects of the faces displayed were very clear and accentuated (e.g., big smiles, frowns), allowing participants to reflect and understand the emotions on display (Carr et al., 2003). As it stands currently, the NMEC model itself has not been extensively scrutinized or tested. However, the model provides a viable explanation of how the mirror neurons' function allows us to empathize with our team-mates' emotions via connections with the limbic system. This is a useful framework to understand the direct perceptual and neuroscientific mechanisms of collective efficacy perceptions, and is something that warrants further exploration regarding this social cognitive process.

As hypothesized, scores for mean collective efficacy and emotional valence were higher in the most confident condition. Difference in collective efficacy scores indicated our experimental manipulation was successful in ensuring participants selected different teams dependent on instructions given. A reciprocal pattern was observed with the eye gaze data, indicating that collective efficacy scores were higher when people fixated on positive images. Similarly, emotional valence of the teams selected by participants supported the greater FD and FC for positive images in the most confident condition. We cannot be certain whether the greater scores in collective efficacy are because participants were instructed to choose their most confident team and therefore felt they should adjust their score accordingly, or because they were influenced by the faces they looked at (i.e., more positive faces) and the teams they selected.

The MANCOVA indicated that baseline self-efficacy scores significantly adjusted the relationship between collective efficacy and how participants were instructed to select their team. Bandura (1997) suggested that individuals first consider their own self-efficacy before making collective efficacy judgements. In the context of this study, this suggests participants had the natural tendency to implicitly consider both how confident they and the displayed faces were, before selecting teammates. For the most confident instruction, as confidence is considered a positive emotion, it is logical that participants would select those with positive faces as vicarious experiences and emotional arousal are important antecedents of self-efficacy (Bandura, 1986). In relation to collective efficacy, we tentatively suggest these two antecedents combine, such that participants assessed “vicarious emotional arousal” (cf. emotional contagion) when making their team selections.

The aim of study two was to explore gaze behavior relating to the proposed action observation that underpins collective efficacy judgements (Bruton et al., 2014). For both familiar and unfamiliar video, individuals fixated on the home team more often and longer than the two other AOI (away team, ball). This only partially supports our hypothesis that the home AOI, which included members of the participants' own team, would be the main AOI for the familiar condition, and that participants would fixate similarly on the home AOI (another sports team from their university) and away AOI (an opposing team in this different sport) for the unfamiliar condition (Bruton et al., 2013). Despite this, the fact that participants in the unfamiliar condition fixated on the away team more frequently (FC) and for longer (FD) compared to the familiar condition still suggests a distinction in visual information sources between these conditions. This may be explained by the need for more information in the unfamiliar condition compared to the familiar video as participants searched for additional vicarious experiences by which to make their judgements, compared to the readily available mastery experiences in the familiar intervention. However, in contrast to our hypothesis that participants would fixate evenly on the home and away teams for the unfamiliar condition, results showed a similar overall bias to the home team. In the unfamiliar video, the home team encompassed another sports team from the host institution performing successfully against an opposing team from another university. Social identity is important for collective efficacy development in sports teams (Fransen et al., 2014, 2015). Therefore, in the unfamiliar condition participants likely identified more with teams affiliated to the host institution and fixated more on them when making collective efficacy judgments.

Our results also supported propositions that video content familiarity is important when manipulating collective efficacy using team-based video. Collective efficacy increased more when individuals observed familiar compared to unfamiliar video. Seeing oneself perform successfully “provides clear information on how best to perform skills, and it strengthens beliefs in one's capability” (Bandura, 1997). It is conceivable observing one's team executing trained skills and tactics provides team-based mastery experiences that reinforce beliefs in the teams' joint capabilities (Bandura, 1994).

Although not to the same magnitude, collective efficacy also increased after observation of unfamiliar team performance. Competitive sports are highly emotive events for spectators (e.g., Raney and Depalma, 2006), meaning performance video of any sports team can evoke emotional responses. We suggest in this instance, participants made favorable social comparisons for transferrable behaviors (e.g., teamwork), leading to increased collective efficacy. In this regard, research indicates individuals spontaneously imagine themselves executing actions when observing others performing actions (Vogt et al., 2013). Given imagery increases efficacy perceptions in sport and exercise settings (e.g., Jones et al., 2002), we tentatively suggest observation of another team performing successfully caused participants to imagine their own team performing successfully.

From a mechanistic perspective, while the NMEC model (Prochazkova and Kret, 2017) provides a useful framework to understand how we observe and process emotional visual content related to collective efficacy, study two provides support that collective efficacy is in part developed through action observation (eye gaze) and the function of the mirror neuron system (Bruton et al., 2016a). Specifically, evidence suggests watching others perform a motor skill (as in study two) innervates our own motor system in a similar manner to which activity would occur if we performed that skill ourselves (Cook, 2016). This is to such an extent that activity in the brain during observation of action is modulated in direct response to the kinematics of that action (Avanzini et al., 2012). Furthermore, an observer will experience a greater motor resonance response when observing movement patterns that exist within their own motor repertoire compared to movements that they have little or no experience in executing (Cook, 2016). Although not directly tested here, given that players from the same team have trained together, follow the same strategic vision, share a common identity, and mostly have the same performance goals, it is plausible that motor resonance would be greatest during observation of players from the same team. Therefore, it is not surprising that participants in study two fixated more often and for longer at the home team and had greater collective efficacy after watching footage of their own “familiar” team.

From a practical perspective, study one suggests emotional management within teams is an important aspect of developing and maintaining collective efficacy. Team members who display positive emotions will contribute positively to collective efficacy. At a team level, the psychologist (e.g., sport, occupational, educational) can educate and raise awareness of the impact of facial emotions and reactions. For example, coaches and managers contribute to the inspiration and motivation of the team (Fletcher and Wagstaff, 2009) and transformational leaders who model behaviors they want to see are an important part of resilient teams (Morgan et al., 2015). Psychologists should therefore encourage positive facial emotions to be displayed by leaders, even as a potential forced response to negative events, as a means to “transmit” collective efficacy across the team. Psychologists can work with individual team members to encourage emotional intelligence and awareness and develop methods of coping or dealing with negative situations that do not rely on the outward expression of negative emotions. Indeed there is strong evidence to suggest that emotional intelligence is an important component of high performing teams (Hodge et al., 2014) and is positively related to coaching efficacy (Thelwell et al., 2008).

Despite the potential importance of our findings, this research is not without limitations. In study one, we used a standardized photo set of emotional faces for our team selection task. While static photos have been used frequently in experimental emotion-based research, in line with the NMEC model, a more dynamic video display might have allowed for greater opportunity for autonomic mimicry to occur. Using video, may have helped delineate some of the marginal differences found here, and in particular might have aided participants understanding of the neutral faces presented. These factors should be considered when interpreting the results of study one, such that with greater opportunity for autonomic mimicry further differences in eye gaze metrics may have been observed between the emotional face AOIs. In study two, we used ecologically valid team-based footage, but focussed on three generalized AOIs based on the assumption participants would “search” the video for either mastery or vicarious experiences (i.e., familiar vs. unfamiliar). Given the complexity of team environments it is likely other non-collective efficacy-based biases might have influenced participant's visual attention. For example, in the unfamiliar video, participants may have attended toward other areas to understand the requirements or rules of the sport before focusing on the actions. It is also possible that the AOI lacked fidelity in terms of the specific information sources used by participants to judge collective efficacy. Making the AOI for “home team” and “away team” more specific in terms of aspects within these AOI (e.g., face, action-relevant limbs, action-irrelevant limbs) might distinguish gaze behavior associated with collective efficacy judgments. This was however not possible in this study given the wide-angle nature of the video footage. Results of study two should be interpreted in such a way that acknowledges the lack of fidelity in measurement, recognizing that the exact areas of interest are as yet imprecise, and that we pose more questions than answers.

There are several future research directions that naturally follow both these studies. First, eye gaze metrics could be used to further examine mechanisms that underpin collective efficacy antecedents. For example, from the perspective of team mastery experiences, vicarious experiences, and non-verbal behaviors, in this study programme we only examined facial emotions and different agents of action (i.e., familiar and unfamiliar teams). In the context of team tasks, much more visual information is available to the observer and future research could extend these studies. For study one, measuring eye gaze during a similar team selection task using whole body pictures or videos with faces included and/or excluded from view would provide a more comprehensive understanding of the role of displayed emotions in collective efficacy perceptions. In regard to study two, we could go beyond a simple “agent” division of AOIs between conditions, using graphic interchange format (GIF) videos to display repeating positive and negative sporting action with a greater number of AOIs. This combined approach might help distinguish when or if emotional vs. action-based perceptions of collective efficacy are more pertinent (e.g., on field vs. off-field).

The results from study two support empirical findings that imagery and observation can be used as interventions for increasing collective efficacy beliefs (Shearer et al., 2007; Bruton et al., 2014). Traditionally, research has focused on action observation (AO) and motor imagery (MI) in isolation, neglecting overlaps and benefits associated with multimodal motor simulation. Recent evidence demonstrates that combined action observation and motor imagery (AO+MI) elicits greater human motor execution network activity and benefits motor processes more than AO or MI independently (Eaves et al., 2016). AO+MI interventions have led to improved performance in sporting tasks when compared to MI (Wright and Smith, 2009), but mixed outcomes were reported for collective efficacy after a 4-week intervention in elite wheelchair basketball teams (Shearer et al., 2009a). Despite this inconclusive finding, AO+MI has received support regarding motor learning and execution (Romano-Smith et al., 2018; Scott et al., 2018; Wright et al., 2018) and warrants further exploration as an efficacy-based intervention.

In conclusion, the two studies presented here are the first to examine emotional and action observation oriented vicarious experiences within the context of collective efficacy. Furthermore, to the best of our knowledge, these are the only two studies that have used gaze behaviors to indicate the possible visual and attentional mechanisms of collective efficacy development. This unique and novel approach has provided a greater depth of knowledge concerning how sport teams (and other groups) develop a sense of confidence. Specifically, in study one, when faced with a choice of available emotions, individuals selected others who display positive emotions in favor of those with neutral or negative expressions. This indicates that in existing teams, facial emotions form an important part of how individuals make collective efficacy judgements about their team. Results from study two are less clear in terms of the significance of the eye gaze metrics but indicate that our eyes are drawn to actions portrayed by players with whom we identify with the most. However, the specific areas of interest when judging collective efficacy while observing team-based actions in this context needs further investigation. Overall the findings have potential for immediate global practical impact for those working with teams in all domains. Further research is needed to understand different sources of information individuals use when observing their team mates vicariously in a subconscious bid to judge collective efficacy.

## Data Availability Statement

The datasets generated for this study are available on request to the corresponding author.

## Ethics Statement

The studies involving human participants were reviewed and approved by The University of South Wales, Faculty of Life Sciences and Education, Faculty Ethics Subgroup, and The University of Roehampton, University Ethics Committee. The participants provided their written informed consent to participate in this study.

## Author Contributions

DS: director of the first research study in paper, and responsible for the overall production of the manuscript. AB: director of the second research study in the paper, wrote the content of study 2 and editorial role in the production of the manuscript. SL, SJ, and ER: data collection and components of data analysis for study 1. MB: data collection and components of data analysis for study 2. RH: advisory roles on all aspects of study 1. MG: advisory roles on all aspects of study 1.

### Conflict of Interest

The authors declare that the research was conducted in the absence of any commercial or financial relationships that could be construed as a potential conflict of interest.
